# Effect of Different Formulations of Magnesium Chloride Used As Anesthetic Agents on the Performance of the Isolated Heart of *Octopus vulgaris*

**DOI:** 10.3389/fphys.2016.00610

**Published:** 2016-12-26

**Authors:** Chiara Pugliese, Rosa Mazza, Paul L. R. Andrews, Maria C. Cerra, Graziano Fiorito, Alfonsina Gattuso

**Affiliations:** ^1^Department of Biology, Ecology, and Earth Sciences, University of CalabriaArcavacata di Rende, Italy; ^2^Association for Cephalopod Research ‘CephRes’Naples, Italy; ^3^Department of Biology and Evolution of Marine Organisms, Stazione Zoologica Anton DohrnNaples, Italy

**Keywords:** Anesthesia, Cephalopods, *Octopus vulgaris*, systemic heart, ethanol, magnesium chloride, Directive 2010/63/EU

## Abstract

Magnesium chloride (MgCl_2_) is commonly used as a general anesthetic in cephalopods, but its physiological effects including those at cardiac level are not well-characterized. We used an *in vitro* isolated perfused systemic heart preparation from the common octopus, *Octopus vulgaris*, to investigate: (a) if *in vivo* exposure to MgCl_2_ formulations had an effect on cardiac function *in vitro* and, if so, could this impact recovery and (b) direct effects of MgCl_2_ formulations on cardiac function. *In vitro* hearts removed from animals exposed *in vivo* to 3.5% MgCl_2_ in sea water (20 min) or to a mixture of MgCl_2_+ ethanol (1.12/1%; 20 min) showed cardiac function (heart rate, stroke volume, cardiac output) comparable to hearts removed from animals killed under hypothermia. However, 3.5% MgCl_2_ (1:1, sea water: distilled water, 20 min) produced a significant impairment of the Frank-Starling response as did 45 min exposure to the MgCl_2_+ ethanol mixture. Perfusion of the isolated heart with MgCl_2_± ethanol formulations produced a concentration-related bradycardia (and arrest), a decreased stroke volume and cardiac output indicating a direct effect on the heart. The cardiac effects of MgCl_2_ are discussed in relation to the involvement of magnesium, sodium, chloride, and calcium ions, exposure time and osmolality of the formulations and the implications for the use of various formulations of MgCl_2_ as anesthetics in octopus. Overall, provided that the *in vivo* exposure to 3.5% MgCl_2_ in sea water or to a mixture of MgCl_2_+ ethanol is limited to ~20 min, residual effects on cardiac function are unlikely to impact post-anesthetic recovery.

## Introduction

Cephalopods are very active creatures (O'Dor et al., 1990; Hanlon and Messenger, 1996; Boyle and Rodhouse, 2005), and have been utilized for more than a century as experimental animals in a broad spectrum of studies in biological sciences (for review see for example: Young, 1985; Hochner et al., 2006; Borrelli and Fiorito, 2008; Hochner, 2012; Huffard, 2013; Ponte et al., 2013; Fiorito et al., 2014). Research required the development of methods to sedate and “anesthetize” these animals (e.g.,: Lo Bianco, 1909; Grimpe, 1928; Young, 1971a). A description of these attempts is already available in Grimpe (1928), and a summary of events occurring during anesthesia with octopus, for example, are found in the classic contribution by Young (1971a).

General anesthesia is necessary to perform surgical or investigative procedures including those required for veterinary and research purposes. Under anesthesia, physiological functions should be maintained as closely as possible to normal ranges and after recovery it is essential that there are no residual deleterious effects.

In the majority of vertebrate species, techniques for general anesthesia are well-developed. Physiological and cellular effects of the agents used for inducing anesthesia are relatively well-understood (Fish et al., 2008; Chau, 2010; see also Sneddon, 2012). In contrast for cephalopods, whilst the potential anesthetic properties of about 20 agents have been explored (for review see for example: Gleadall, 2013; Fiorito et al., 2015), few studies regarding the physiological effects on the animals, including the cardiac ones, are available (review in: Andrews et al., 2013).

The inclusion of cephalopods in Directive 2010/63/EU regulating the use of animals in scientific research (European Parliament and Council of the European Union, 2010 for review see: Smith et al., 2013; Fiorito et al., 2015) has prompted reconsideration of the criteria for general anesthesia in cephalopods and review of their physiological and pharmacological effects (see review in: Andrews et al., 2013; Fiorito et al., 2014, 2015).

Magnesium chloride (MgCl_2_) has been used as an anesthetic agent for cephalopods more than 75 years ago (e.g., Mitolo, 1938). However it is only after Messenger et al. that the use of MgCl2 increased. Until then urethane (i.e., ethyl carbamate) was the most widely used anesthetic agent for surgery (e.g., brain lesions) in octopus and other cephalopods (e.g.,: Sanders and Young, 1940; Boycott and Young, 1950, 1955; Young, 1971a; for review see also Gleadall, 2013). Ethyl carbamate was abandoned because of its elevated carcinogenicity (Nomura, 1975).

In various formulations MgCl_2_ has been used to anesthetize species representative of the three major orders of cephalopods, i.e., Sepiida, Teuthida, and Octopoda, such as *Sepia officinalis, Loligo forbesi, Dorytheutis pealei, Sepioteuthis sepioidea, Ilex illecebrosus, Octopus vulgaris*, and *Eledone cirrhosa* (review in Fiorito et al., 2015). Magnesium chloride, either in sea water or as a mixture of sea water and distilled water, fulfills the criteria commonly used for assessing general anesthesia in cephalopods (Andrews and Tansey, 1981; for review and discussion see: Andrews et al., 2013; Gleadall, 2013; Fiorito et al., 2015), in particular: skin pallor (mantle and arms) and loss of texture, loss of arm muscle tone and sucker adhesiveness, loss of the righting reflex, absence of a response to a noxious mechanical stimulus applied to the arms or mantle and marked suppression of ventilation.

The systemic heart rate in *O. vulgaris* anesthetized with 3.5% magnesium chloride in sea water is reportedly “very low” (Fiorito et al., 2015), and is “slow” in *O. vulgaris* anesthetized with a mixture of magnesium chloride and ethanol (Grimaldi et al., 2013); in neither case was quantitative data presented to support the comments. The reported cardiac suppressive effect of MgCl_2_ (± ethanol) in *O. vulgaris* is consistent with the effects of magnesium ions on the heart in mammals, including humans (Engbaek, 1952).

According to Young, the “heart beat stops” when using urethane as an anesthetic agent (Young, 1971a) and again, ≪this makes operation simpler because of absence of bleeding, but limits its duration (using urethane) to 15–20 min≫ (Young, 1971a, p. 642).

A marked suppression of heart rate, will inevitably be associated with a fall in cardiac output and blood pressure, reduced tissue perfusion and ischemia, with the brain likely to be particularly vulnerable. Additionally, a profound fall in blood pressure may contribute to central nervous system depression including suppression of ventilation driven from the posterior sub-oesophageal lobes (for a functional description of the octopus brain see Young, 1971b) further exacerbating the tissue ischaemia. Cardiovascular effects of anesthetic agents could, by reducing brain perfusion, contribute to loss of consciousness.

In view of the widespread use of magnesium chloride as an anesthetic agent in cephalopods (Fiorito et al., 2015), there is a necessity to understand its actions in the various formulations utilized, on the physiological performance of the systemic heart of *O. vulgaris*.

The *in vitro* working preparation of the systemic heart is able to generate physiological values of cardiac output, output pressure, ventricle work, and power in various cephalopod preparations, e.g.,: *S*. *officinalis* (Kling and Jakobs, 1987; Jakobs and Schipp, 1992; also perfused *in situ* in MacCormack et al., 2016), *E. cirrhosa* (Smith, 1981), and *O. vulgaris* (Foti et al., 1985; Houlihan et al., 1987; Agnisola et al., 1989; Agnisola and Houlihan, 1994).

The effects on cardiac performance of three different magnesium chloride formulations were investigated in this study by using (i). 3.5% MgCl_2_ made up in sea water, (ii) 3.5% MgCl_2_ made up in a 1:1 mixture of sea water:distilled water, and (iii) 1.12% MgCl_2_ and 1% ethyl alcohol mixture dissolved in sea water. These have been utilized in a large number of studies as reviewed by Fiorito et al. (2015).

The 3.5% MgCl_2_ formulation (1:1 mixture of sea water:distilled water, see Pagano et al., 2011) utilized here is a minor modification of the original 3.75% MgCl_2_ utilized by Messenger et al. (1985).

The *in vitro* systemic heart preparation was used to address two questions. First, does exposure to the various formulations of MgCl_2_ *in vivo* have residual effects on the heart and, if so, could these compromise recovery from anesthesia? Second, what direct effects do the various formulations of MgCl_2_ and ethanol have on cardiac function?

Our results suggest that although MgCl_2_ formulations have some direct effects on the heart (*in vitro* studies), *in vivo* they are suitable for inducing anesthesia, provided exposure time is carefully controlled.

## Materials and methods

### Animals

*O. vulgaris* of both sexes (males: *N* = 32; females: *N* = 18; body weight, mean ± SEM: 574 ± 25 g) were caught in the Bay of Naples (Italy) by local fishermen and transported to Arcavacata (Cosenza, Italy) according to the best-practice for long-duration transportation (Byrne et al., 2004; review in Fiorito et al., 2015).

Octopuses were housed individually in opaque tanks (40 × 50 × 80 cm) with circulating sea water (18–22°C) and maintained according to the best practice (e.g., Agnisola et al., 1996) for up to 3 days prior to killing. Animals were randomly assigned to the various experimental groups.

Age of octopuses cannot be estimated in live individuals, but assessed *post-mortem*, and does not appear to correlate with body weight (Canali et al., 2011). Therefore, information on age of the octopuses was not available at the time the animals were assigned to experimental groups.

### Regulatory considerations

Research studies involving “live cephalopods” within the EU are covered by Directive 2010/63/EU (European Parliament and Council of the European Union, 2010) and its subsequent transposition into legislation of Member States (see also: Smith et al., 2013; Fiorito et al., 2015).

The studies reported here were performed before Italy transposed the Directive (March 2014). In addition, these experiments required killing the animal for the sole purpose of removing tissue. Therefore, they fall outside the scope of the Directive 2010/63/EU, provided that an approved method of killing is used (see below). Ethical review of the experiments at institutional level was undertaken at the study site (Cosenza, Italy).

This study adhered to the *ethos* of the Directive which specifies that killing must be performed by an “adequately educated and trained person” using an approved method (European Parliament and Council of the European Union, 2010). Although cephalopods are included in the Directive, no specific recommendations of methods for killing are listed in Annexe IV. In view of this we have applied the general principles outlined in Annexe IV (1a) to cephalopods (Andrews et al., 2013; Fiorito et al., 2015) that, when killed the animal should be unconscious, should remain unconscious until death ensues and is confirmed.

In all experiments included here the animals fulfilled the criteria for general anesthesia (as mentioned above, and see: Andrews and Tansey, 1981; Andrews et al., 2013; Gleadall, 2013; Fiorito et al., 2015) when killed.

### Killing method

Depending upon the protocol, the animals were exposed to one of the following treatments:

- **MgCl**_2_**:** Immersion for 20 min in 2 L of 3.5% magnesium chloride dissolved in sea water (sw) at room temperature (18–21°C). At this time the animals are immobile, pale, lack a righting reflex, are unresponsive to handling, and ventilation is suppressed or absent (see review of criteria in Fiorito et al., 2015).
- **MgCl**_2_ **(1:1):** Immersion for 20 min in 2 L of 3.5% magnesium chloride dissolved in a mixture of sea- and distilled-water (1:1, sw:dw as in Pagano et al., 2011). After 20 min the appearance of the animal is similar to the above description and to the original one in Messenger et al. (1985). Note that similar to Pantin (1946), Messenger et al. (1985) mixed 7.5% MgCl_2_ dissolved in distilled water with an equal volume of sw to achieve a final concentration of 3.75% MgCl_2_.
- **Mix:** Immersion in a 2 L mixture of magnesium chloride (1.12%) and ethanol (1%) in sea water. Exposure was either for 20 min **(Mix 20**′**)** or 45 min **(Mix 45**′**)** to match the times used by some authors for achieving anesthesia in *O. vulgaris* for neurophysiological studies (i.e., 55 mM MgCl_2_ and 1% ethanol in sw: (Shomrat et al., 2008)) or other purposes (Pagano et al., 2011). Exposure times of 25–45 min to a mixture of MgCl_2_ (1.12%) and ethanol (1%) have also been used for induction of deep anesthesia in *O. vulgaris* (Shomrat et al., 2008, 2011).
- **Hypothermia**: Immersion in 2 L of sea water at 4°C for 5–10 min. Profound cooling of Mediterranean *O. vulgaris* has been used to “anaesthetize” animals when the use of a chemical agent could compromise the experimental outcome (Andrews et al., 1981). Although there is debate (Gleadall, 2013) about the exact effects of cooling these animals well-outside their normal thermal range (see also discussion in Agnisola et al., 1996), cooling has been reported to produce a state comparable to the one induced by the anesthetic agents urethane and ethanol (Andrews and Tansey, 1981).

After treatments the brain was destroyed to complete killing, as described in Directive 2010/63/EU Annexe IV (2b) (see recommendations in: Andrews et al., 2013; Fiorito et al., 2015).

### Systemic heart isolation

Immediately after brain destruction, the systemic heart was isolated according to Foti et al. (1985) and Houlihan et al. (1987). The heart dissection was performed at 4°C and took ~ 15 min.

In *O. vulgaris*, blood enters the heart through two auricles and leaves the ventricle through three arteries: the dorsal (cephalic) aorta and the abdominal and gonadial arteries. A rich network of coronary veins is present on the surface of the heart which drain the blood directly from the ventricular lumen during ventricular systole (Agnisola et al., 1990). Both the auricles and the dorsal aorta were cannulated while the gonadial and abdominal arteries were ligatured at their base. The outflow from the coronary veins passed out into the chamber and was collected by an overflow system. The fusiform ganglion was removed.

The heart was connected to the perfusion apparatus where the two auricles received perfusion fluid at the same controlled input pressure. The perfusion solution (PS) contained filtered sea water containing 2.78 mM anhydrous glucose, and was gassed with 99.5% oxygen and 0.5% carbon dioxide. The pH of the perfusion medium was 8.0 (as in: Houlihan et al., 1987; Agnisola et al., 1989; Agnisola and Houlihan, 1994) and is close to the pH of *O. vulgaris* haemolymph (pH = 7.8 ± 0.1; D'Aniello et al., 1986).

The basal perfusion conditions were chosen to reproduce *in vivo* resting hemodynamic parameters as previously reported (Agnisola et al., 1989; Agnisola and Houlihan, 1994). The preload (input pressure) was adjusted to obtain a stroke volume (SV) of 0.7–0.8 ml/g of ventricular weight, a value that is approximately physiological for resting animals of a similar size to those used here (Wells et al., 1987). The diastolic output pressure was always set at 2 kPa above the preload which corresponds to the diastolic aortic pressure in resting animals (Wells and Smith, 1987). Experiments were performed at room temperature (18–21°C). The heart beats spontaneously and does not require pacing at this temperature. Under these conditions, aortic pressure, heart rate, aortic, and coronary flow were measured for 15–20 min to assess their stability; if parameters had not stabilized within the range established in previous studies the experiment was not continued. About 15% of hearts failed to meet the criteria for stabilization, but there was no obvious relationship between body weight, sex, and anesthetic protocol and failure to stabilize.

### Measurements and calculations

Preload and afterload (in kPa) were defined as the mean input and output pressures, respectively. The pressure measurements were referred to the level of perfusate in the perfusion chamber and corrected for cannulae resistance. Pressures and heart rate (HR, beats/min) were measured through two MP-20D pressure transducers (Micron Instruments, Simi Valley, CA) connected to a PowerLab data acquisition system and analyzed using Chart software (AD-Instruments, Ugo Basile, Comerio, Italy).

Other cardiac parameters were measured as follows:

**Cardiac output** (***Q***, ml/min/g) and **Coronary output** (**CorO**, ml/min/g) were derived from the dorsal aorta outflow and coronary vein outflow, respectively, collected over 1 min, and weighed. Values were corrected for fluid density and normalized per gram ventricle wet weight.
**Stroke volume** (**SV**, ml/g) was calculated from ***Q***/**HR** and used as an index of contractility.
**Stroke work** (**SW**, mJ/g) was calculated as (afterload-preload) × **SV**/ventricular wet weight.
**Power output** (**PO**, mW/g) was calculated as (afterload-preload) × ***Q***/60

The separation between aortic and coronary output is necessary because during contraction, a proportion of the total cardiac output is collected in the coronary veins passing through the walls of the heart (Foti et al., 1985; Houlihan et al., 1987; Agnisola and Houlihan, 1994). Thus, contraction of the isolated ventricle resulted in ejecting flow through the cannulated aorta (i.e., aortic output) and through the walls of the heart, into the cut coronary veins (i.e., coronary output). In figures ***Q***, SV, and SW are related to aortic output.

### Experimental protocols

#### Protocol I: Frank–starling curves

Following isolation of the systemic heart from animals (*N* = 27) immersed in the different magnesium formulations [*N* = 5 for MgCl_2_; *N* = 5 for MgCl_2_ (1:1); *N* = 5 for Mix 20′; *N* = 6 for Mix 45′] or hypothermia [*N* = 6 (4°C)], Frank–Starling curves were generated to assess the effect of these treatments on the cardiac sensitivity to increased preloads. The Frank-Starling mechanism (heterometric cardiac regulation) is a property of the myocardium to respond to increased venous return (preload) by a more forceful contraction (contractility) of its lengthened fibers, thus increasing stroke volume (SV) and hence cardiac output (***Q***).

After an initial period of stabilization (15–20 min) at baseline conditions, the input pressure was increased stepwise in 0.1 kPa increments until the maximal cardiac output was reached. Each pressure increment was maintained for 5 min during which cardiac parameters were evaluated.

#### Protocol II: anesthetic concentration-response curves

The systemic heart was isolated from animals (*N* = 23) killed following hypothermia, in order to avoid possible interference with the following treatment. After stabilization of cardiac parameters (15–20 min) during which the hearts were perfused with PS, cumulative concentration-response curves were generated to evaluate the action of the different anesthetic regimes on cardiac function with the agents delivered in PS. The formulations tested were: MgCl_2_ (*n* = 7) and MgCl_2_ (1:1, *n* = 5) starting from 0.25%, and increasing to: 0.5, 1, and 2%; Mix (*n* = 6) at 0.14/0.125, 0.28/0.25, 0.56/0.5, and 1.12/1%. In addition, ethanol alone (*n* = 5) at increased concentrations (0.125, 0.25, 0.5, and 1%) was also tested to separate the effects of MgCl_2_ from ethanol in the mixture. Each concentration was tested for 10–15 min.

### Chemicals, solutions, and osmolality measurements

The following chemicals were used: Ethanol (99%, APPLICHEM, CAS Number 64-17-5); Glucose (SIGMA, CAS Number: 50-99-7); Magnesium chloride hexahydrate (SIGMA ALDRICH, CAS Number: 7791-18-6).

The osmolality of the sea water, perfusion medium and the anesthetic formulations used for the experiments was measured using an autocal osmometer (Roebling) according to recommendations for the use of osmometry methods for biological samples (Sweeney and Beuchat, 1993). For each solution four samples were measured (at 20°C) in triplicate.

### Statistics

Results are expressed as mean ± SEM of percentage changes obtained from individual experiments unless otherwise stated. Statistical analysis was performed on raw data (not %) following Zar (1999). All data were tested for normality. We utilized repeated-measures ANOVA followed by Bonferroni's Multiple Comparison test whenever appropriate. Multivariate Analysis of Variance (MANOVA) was utilized to test effects of different treatments for Frank-Starling curves. Mixed Model ANOVA was utilized as method for testing effects of repeated-measures when the sample numbers were not matched for all treatments/parameters. Differences were considered statistically significant at *p* < 0.05. For all statistical analyses we used SPSS (rel. 14.0, SPSS Inc—Chicago, 2005).

## Results

### Osmolality measurements

Osmolality values of the solutions used for *in vivo* (utilized to anesthetize the animals) and *in vitro* (by perfusion) studies are reported in Table 1. The osmolality of sea water was 1125.25 ± 1.39 mOsm/Kg (*n* = 4) and the osmolality of the perfusion solution (PS: Sea water+glucose) was 1139.67 ± 1.17 mOsm/Kg (*n* = 4). In our conditions, all solutions had higher osmolality values than sea water and PS, with the exception of MgCl_2_ (1:1) where the osmolality was about 10% lower than either PS or sea water. It is interesting to note that haemolymph in Mediterranean *O. vulgaris* has an osmolality of ~1300 mOsm/Kg or higher (D'Aniello et al., 1986; Wells and Wells, 1989).

**Table 1:** **Osmolality of the anesthetic solutions utilized in this study**.

**Solutions**		**Osmolality (mOsm/Kg)**					
MgCl_2_	**%**	*0.25*	*0.5*	*1*	*2*	*2.5*	*3.5*
		1152.67 ± 2.79	1186.17 ± 1.70	1254 ± 2.47	1410.67 ± 3.14	1476.33 ± 4.94	1605.92 ± 3.31
MgCl_2_(1:1)	**%**	*0.25*	*0.5*	*1*	*2*	*2.5*	*3.5*
		585.33 ± 1.10	617.83 ± 1.80	694 ± 3.83	831.58 ± 1.94	887.33 ± 0.99	1009.92 ± 3.93
Mix^1^	**%**	*0.14/0.125*	*0.28/0.25*	*0.56/0.5*	*1.12/1*		
		1163.67 ± 1.39	1212.67 ± 0.94	1310 ± 2.56	1481.42 ± 2.72		
Ethanol	***%***	*0.125*	*0.25*	*0.5*	*1*		
		1144.17 ± 1.51	1169.5 ± 1.53	1215.58 ± 2.85	1313.33 ± 1.67		

*Values are derived from measurements from three replicates of four samples for each solution. For each formulation the first row refers to the concentration (%, in italics) of substances utilized and the second to the mean (± SEM) osmolality calculated for each solution. 1. for all cases, % of MgCl_2_ precedes the % value of EtOH utilized*.

### Baseline hemodynamic parameters in the isolated heart

The baseline hemodynamic parameters in the spontaneously beating isolated hearts from animals with the five different pre-isolation treatments are summarized in Table 2. Irrespective of the different anesthetic treatments (including hypothermia), at baseline conditions the perfused hearts were able to produce physiological values of stroke volume and to work at hemodynamic loads comparable with those reported by other authors for *in vitro* preparations (Agnisola et al., 1989; Agnisola and Houlihan, 1994). No differences were detected in baseline parameters of hearts with different treatments, with exception of afterload pressure and CorO (Table 2). Although the afterload values were in the physiological range (see as reference: Wells et al., 1987; Wells and Smith, 1987) differences emerged only when comparing MgCl_2_ vs. hypothermia and MgCl_2_ vs. Mix 45′ (Table 2). In addition, the coronary outputs were only significantly different when comparing hypothermia vs. MgCl_2_ (1:1) or Mix 20′ (Table 2).

**Table 2:** **Baseline hemodynamic parameters of *O. vulgaris* isolated hearts after stabilization**.

**Anesthetic treatment**	**Preload (kPa)**	**Afterload (kPa)**	**HR (beats/min)**	***Q*** **(ml/min/g)**	**CorO (ml/min/g)**	**SV (ml/g)**	**SW (mJ/g)**	**PO (mW/g)**
Hypothermia (*N* = 6)	0.33 ± 0.04	24.13 ± 0.55^*^	23.50 ± 1.86	17.99 ± 1.45	3.09 ± 0.45^*^	0.77 ± 0.03	1.79 ± 0.08	0.69 ± 0.05
MgCl_2_(*N* = 5)	0.40 ± 0.07	29.22 ± 0.96^*^	29.22 ± 3.30	20.91 ± 2.28	4.40 ± 0.40	0.69 ± 0.05	1.95 ± 0.18	0.91 ± 0.05
MgCl_2_ (1:1) (*N* = 5)	0.32 ± 0.04	27.07 ± 1.07	28.84 ± 2.22	21.79 ± 2.71	4.90 ± 0.29^*^	0.76 ± 0.07	1.97 ± 0.19	0.95 ± 0.11
Mix 20′(*N* = 5)	0.37 ± 0.05	24.85 ± 1.75	29.59 ± 3.11	19.45 ± 1.33	4.75 ± 0.26	0.68 ± 0.06	1.63 ± 0.11	0.77 ± 0.05
Mix 45′ (*N* = 6)	0.49 ± 0.03	23.08 ± 0.98^*^	29.00 ± 4.05	19.39 ± 3.70	5.04 ± 0.48^*^	0.68 ± 0.08	1.51 ± 0.20	0.70 ± 0.11

Values are plotted as mean ± SEM (N = number of animals per treatment). HR, heart rate; **Q**, cardiac output; CorO, coronary output; SV, stroke volume; SW, stroke work; PO, power output. One-way ANOVA (Afterload): F_(4, 22)_ = 5.07, p = 0.005, after Bonferroni's Multiple Comparison Test (^*^): MgCl_2_ vs. hypothermia, p = 0.031; MgCl_2_ vs. Mix 45′, p = 0.006. One-way ANOVA (CorO): F_(4, 22)_ = 4.17, p = 0.012, after Bonferroni's Multiple Comparison Test (^*^), hypothermia vs. MgCl_2_ (1:1), p = 0.031; hypothermia vs. Mix 45′, p = 0.012; hypothermia vs. Mix 20′, p = 0.054 (marginally significant).

### Frank–starling curves

**Figure 1:**
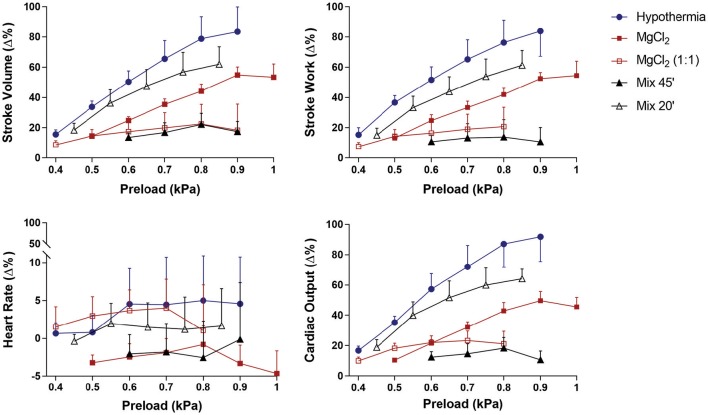
**Effect of preload increase on stroke volume (SV), stroke work (SW), heart rate (HR), and cardiac output (*Q*) in the isolated *Octopus vulgaris* systemic heart (*n* = 5, 6 each treatment) following removal under hypothermia (4°C), MgCl_2_, MgCl_2_ (1:1), and MgCl_2_+EtOH mixture following 20 (Mix 20′) and 45 (Mix 45′) minutes of exposure**. Results are expressed as mean ± SEM of the percentage (%) change from baseline (see Table 2). Repeated-measures ANOVA revealed no significant differences among treatments, but treatments × preload effects were significant with the exception of HR (SV: *F*_(4, 16)_ = 2.16, *p* = 0.121; treatments × preload *F*_(24, 96)_ = 3.29, *p* < 0.001; SW: *F*_(4, 16)_ = 2.62, *p* = 0.074; treatments × preload *F*_(24, 96)_ = 4.42, *p* < 0.001; HR: *F*_(4, 16)_ = 0.84, *p* = 0.518; treatments × preload *F*_(24, 96)_ = 0.31, *p* = 1.000; ***Q***: *F*_(4, 16)_ = 0.36, *p* = 0.831; treatments × preload *F*_(24, 96)_ = 6.14, *p* < 0.001. MANOVA was utilized to evaluate pairwise differences in preload values between MgCl_2_ (1:1) and (Mix 45′) and the other curves (see text for details). The largest response in terms of ***Q***, SV, and SW was obtained with hypothermia followed by treatment with Mix 20′ and MgCl_2_. Hypothermia, ***Q*** = 91.8 ± 17.2%; SV = 83.4 ± 14.9%; SW = 83.9 ± 15.9%; MgCl_2_, ***Q*** = 49.6 ± 4.3%; SV, 54.9 ± 3.7%; SW = 52.5 ±2.9%; Mix 20′, ***Q*** = 64.1 ± 7.4%; SV = 61.9 ± 11.5%; SW = 56.5 ± 10.5%. MgCl_2_ (1:1) and Mix 45′ showed the worst response revealed by an impaired ability to respond to preload increases [MgCl_2_ (1:1): ***Q*** = 23.3 ± 6.4%; SV = 22.5 ± 11.5%; SW = 20.8 ± 11.4%; Mix 45′, ***Q*** = 22.9 ± 4.0%; SV = 22.2 ± 7.4%; SW = 13.9 ± 11.5%].

Frank-Starling curves obtained from cardiac preparations of animals treated with MgCl_2_, MgCl_2_(1:1), Mix 20′, Mix 45′, and hypothermia showed that, with increasing preload, a significant raise in ***Q***, SV, and SW occurred (Figure 1). The preload increases did not produce a significant change in the heart rate in any of the treatments, thus the increases in cardiac output were due solely to an increase in stroke volume.

The Frank-Starling curves for the individual treatments are plotted in Figure 1. Hearts isolated from animals under either hypothermia or MgCl_2_in sea water endured a higher number of pre-load increments (6 increments) before reaching the plateau of the Frank-Starling curve (i.e., the optimal preload at which point ***Q*** remains constant) followed by Mix (20′) and MgCl_2_ (1:1) (5 increments), and Mix 45′ (4 increments).

The individual Frank-Starling curves (Figure 1) for each pre-isolation treatment revealed that over a similar pressure range the curves for MgCl_2_(1:1) and Mix 45′ were flatter than those for all other pre-isolation treatments. This is confirmed by the maximum values reached for all cardiac functions parameters (see legend in Figure 1). At the maximal input pressure used (until there was no further increase in ***Q***), the largest response in terms of ***Q***, SV, and SW was obtained with hypothermia followed by treatment with Mix 20′ and MgCl_2_. MgCl_2_(1:1), and Mix 45′ showed the worst response revealed by an impaired ability to respond to preload increases (Figure 1).

Repeated-measures ANOVA confirmed this view, revealing no significant differences between any of curves for each treatment for any of the parameters considered (SV, SW, HR, and ***Q***, see Figure 1), but also highlight a significant treatment^*^preload effect for all parameters considered (*p* < 0.001), with the exception of HR (*p* = 0.999), thus confirming that the hearts all responded to increases in preload. Inspection of the Frank-Starling curves (Figure 1) revealed that hearts isolated under hypothermia, Mix 20′ and MgCl_2_ were all capable of increasing stroke volume by ~50% or more, whereas this was not the case for the MgCl_2_ (1:1) and Mix 45′ hearts. Bonferroni's Multiple Comparison Tests after Multivariate Analysis of Variance (MANOVA) confirmed this view. Significant differences in the preload-stroke volume relationship were observed only for hypothermia vs. Mix 45' (preload values 0.6, 0.8, 0.9 kPa, *p* < 0.05) and for hypothermia vs. MgCl_2_(1:1) only at the two highest preloads (*p* < 0.05). A similar view emerged considering stroke work where significant differences resulted for hypothermia vs. Mix 45′ (*p* < 0.05) and MgCl_2_ vs. Mix 45′ (*p* < 0.05, only at the last preload value). No other significant differences emerged when considering other parameters.

Figure 1 also shows that the Frank-Starling curves for hypothermia, Mix 20′ and MgCl_2_ are parallel to each other; in addition, the responses of the hearts isolated under MgCl_2_(1:1) and Mix 45′ are similar to each other. In the latter the preload-stroke volume relationship was shallow in comparison to that in hearts isolated under hypothermia, Mix 20′ or MgCl_2_. These differences in slope are also reflected in calculations of the change in stroke volume per kPa change in input pressure (δ kPa) using the values from the individual Frank-Starling curves; for hypothermia (*n* = 6): 2.01 ± 0.21 ml/g/kPa; for MgCl_2_ (*n* = 5): 1.4 ± 0.10 ml/g/kPa; for MgCl_2_ (1:1; *n* = 5): 1.01 ± 0.16 ml/g/kPa; for Mix 20′ (*n* = 5): 1.52 ± 0.14 ml/g/kPa; for Mix 45′ (*n* = 6): 1.08 ± 0.18 ml/g/kPa.

### Anesthetic concentration-response curves

**Figure 2:**
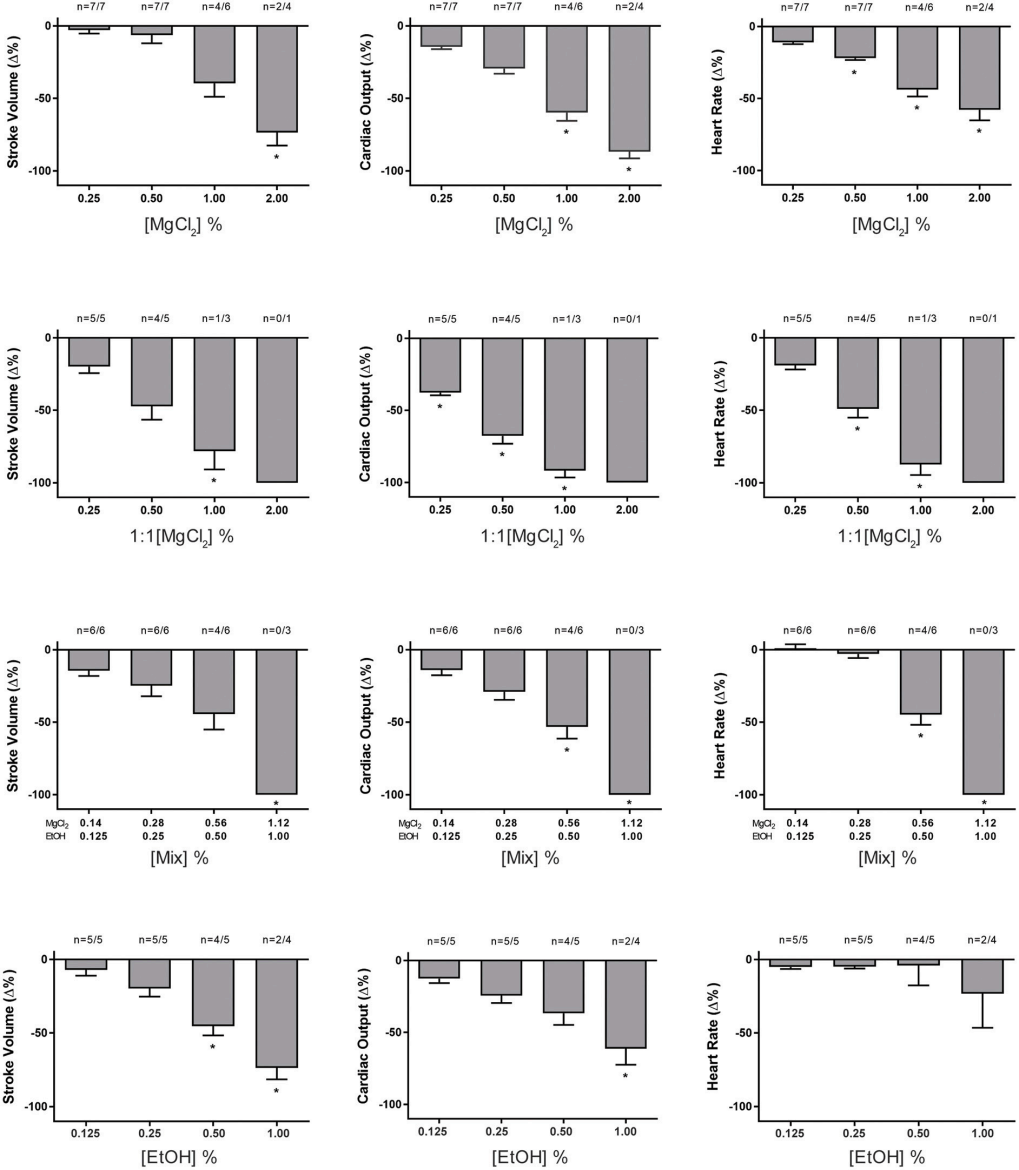
**Effect of increasing concentrations of MgCl_2_, MgCl_2_ (1:1), Mix, and ethanol on the percentage change from baseline values in heart rate (HR), stroke volume (SV), and cardiac output (*Q*); values are presented as mean ± SEM percentage (%) change**. The number of hearts (n, beating/tested) are indicated above each column. Significant differences are marked by (^*^, *p* < 0.05). Note that at the same concentration of MgCl_2_, the effects of the 1:1 formulation are greater than the sea water formulation. In addition, whilst both Mix and ethanol affect the stroke volume and cardiac output, the effects of ethanol alone on heart rate are less marked (0.5% and 1%) in comparison to the same concentration of ethanol mixed with magnesium chloride. See text for details.

Perfusion with increasing concentrations of different anesthetic solutions was carried out on systemic hearts removed under hypothermia and the effects on HR, SV, and ***Q*** are plotted in Figure 2. All anesthetics, except ethanol, induced bradycardia in a concentration-related manner. In particular, MgCl_2_ and Mix significantly reduced HR, ***Q***, and SV (*p* < 0.01 after Mixed Model ANOVA, except HR with EtOH) at the higher concentrations tested (Figure 2).

**Figure 3:**
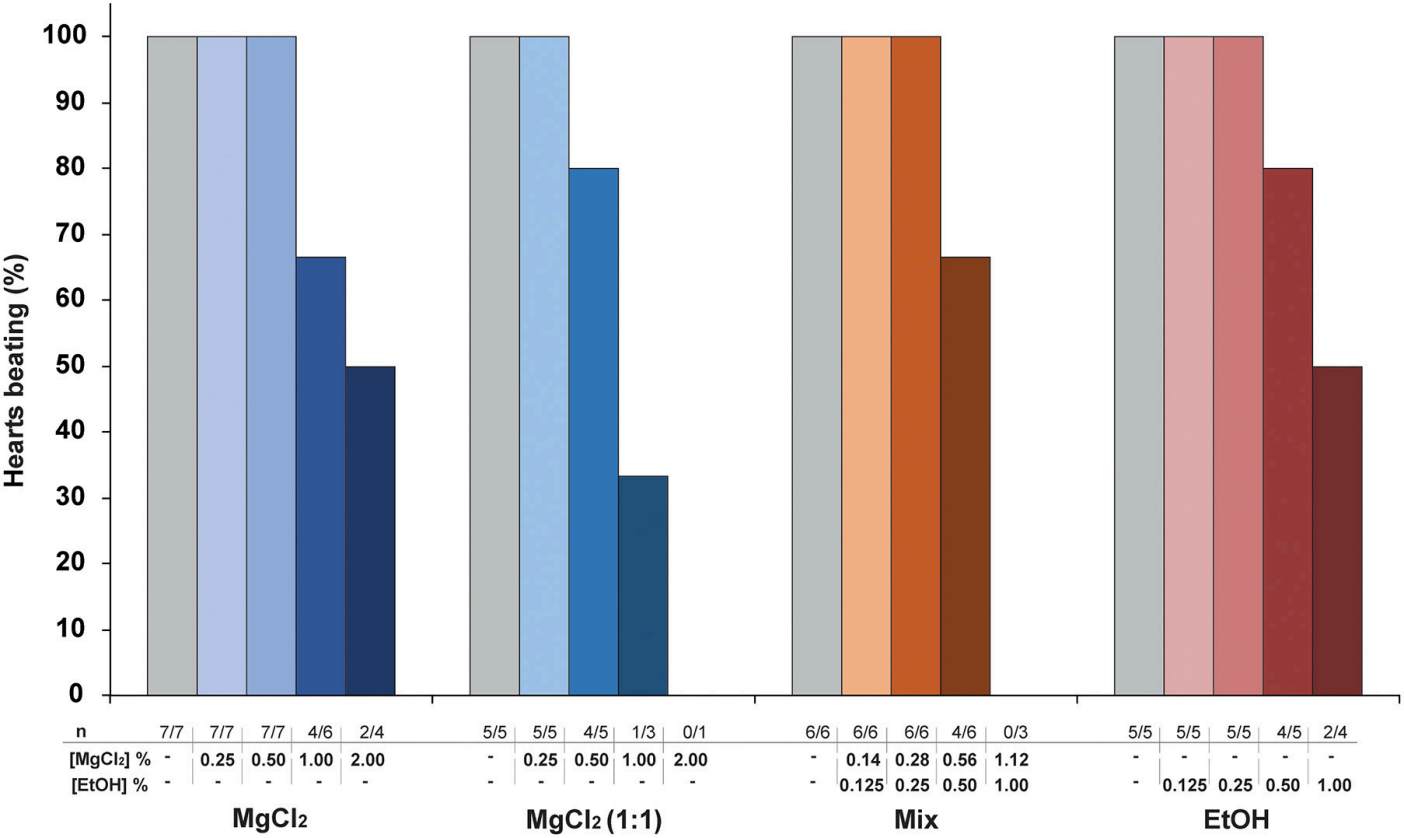
**The effect of cardiac perfusion with increasing concentrations of anesthetic solutions on the incidence of spontaneous beating in hearts isolated under hypothermia (see Methods for details)**. The number of hearts treated is lower at the higher concentrations, as fewer studies were conducted at concentrations above the threshold where arrest was first observed.

MgCl_2_ (1:1) appears to be a more potent cardio-suppressive agent than either MgCl_2_ or Mix because it induced a significant reduction of cardiac parameters starting at a lower MgCl_2_ concentration. All anesthetic solutions were able to produce cardiac arrest at the higher concentrations used (Figure 3) with the incidence of arrest lowest with MgCl_2_ and ethanol alone. In all cases the heart could be restarted in <10 min, when re-perfused with PS alone (data not shown).

## Discussion

The results provide insights into the physiological effects on the systemic heart of magnesium chloride formulations used for anesthesia of *O. vulgaris*. The effects of the anesthetic formulations on heart rate and contractility *in vitro* have implications for their use as common anesthetic agents in cephalopods and contribute to discussions of their anesthetic effects (Andrews et al., 2013; Gleadall, 2013; Fiorito et al., 2015). The two questions about the cardiac effects of MgCl_2_ formulations posed in the introduction will be discussed separately before considering the possible mechanism and the potential implication of these findings for cephalopod anesthesia.

### Does exposure to the various formulations of MgCl_2_ *in vivo* have residual effects on the heart *in vitro*?

#### Basal conditions

Baseline values of cardiac function from both hypothermic and magnesium-treated animals (Table 2) are similar to those reported for isolated hearts perfused with oxygenated saline (Agnisola and Houlihan, 1991). Perfusion conditions replicated physiological resting afterload and stroke volume values as previously reported (Agnisola et al., 1989, 1994). All preparations were stable for 70–100 min, a time comparable to Agnisola et al. (1994). Other authors reported a shorter period of viability (about 50 min, Houlihan et al., 1987), followed by a notable decline in cardiac output, probably due to the higher input pressures used (0.5–2.0 kPa, Houlihan et al., 1987).

*In vitro* baseline input pressures were higher than those reported *in vivo* (0.05–0.25 kPa in the efferent branchial vessel, Wells and Smith, 1987), but similar to those previously reported *in vitro* in *O. vulgaris* (0.49 kPa, Smith, 1981; Houlihan et al., 1987; 0.23 kPa, Agnisola et al., 1989). The mean afterload was within the physiological range: resting values of 2.0 kPa in diastole or 3.5 kPa in systole are typical of animals in the 400–1000 g body weight range (Wells et al., 1987; Wells and Smith, 1987). The cardiac output was below the *in vivo* value (44 ml/min/kg, Houlihan et al., 1986), but comparable to those reported *in vitro* by Agnisola et al. (1994).

Linkage between cardiac output and coronary flow in the *in vitro O. vulgaris* heart was reported by Foti et al. (1985); during systole a proportion of the total cardiac output is collected in cut coronary veins. In our preparation, the amount of the aortic output entering the coronary system ranged from about 17% (hypothermia) to 26% (Mix 45′). These values are lower than those reported (e.g.,: 41.5 ± 3.6% in Houlihan et al., 1987) using higher values of input and output pressures of 20 and 40 cmH_2_O, respectively. However, they are comparable to those obtained by Agnisola et al. (1989, 1994) where similar and more physiological loading pressures were used.

As expected from other *in vitro* studies where extrinsic neural inputs are removed, the baseline heart rate was lower than the *in vivo* range of 35–45 bpm (Wells, 1979, 1980; Wells and Smith, 1987; Fiorito et al., 1998), but in the same range as those reported by other authors *in vitro*, i.e., 30 ± 0.7 bpm (Houlihan et al., 1987), 34 ± 1 bpm (Agnisola et al., 1989) and 26.3 ± 0.8 bpm (Agnisola et al., 1994).

Overall, with regards to baseline hemodynamic parameters (Table 2) there was little to differentiate the effect of various pre-treatments, with the exception of the hearts removed from animals anesthetized with 3.5% MgCl_2_ that produced a significantly higher afterload compared to hearts isolated under hypothermia and the significantly higher coronary flow in hearts isolated under MgCl_2_ (1:1) and Mix 45′ compared to hypothermia.

#### Frank-starling curves

In terms of the responsiveness of cardiac performance (functional state of the heart as a pump in relation to contractility, work, and heart rate) to input pressures (Frank-Starling response), our preparation performed in a similar way to previous *in vitro* studies on octopus (Smith, 1981; Foti et al., 1985; Houlihan et al., 1987); cardiac output has been shown to be primarily affected by changes in stroke volume, with input pressure having little effect on heart rate. Regarding the heart rate-preload relationship, it has been reported that in the *in vitro* spontaneously beating systemic heart of octopus the rate is directly affected by input pressure when the latter is above the physiological range as reported originally by Fredericq (1878, 1914), and later by Smith (1981) and Foti et al. (1985). In contrast, the dependence of heart rate on the input pressure is irrelevant when physiological values are used (present study and Houlihan et al., 1987). In fact, here and irrespective of the anesthetic pre-treatment, the heart rate did not change when preload increased.

As expected, increased preload produced an enhanced stroke volume and hence cardiac output; however, baseline conditions and the Frank-Starling response were only affected to a limited extent by different anesthetic pre-treatments. In particular, systemic hearts removed from animals treated with hypothermia exhibited the best cardiac performance (as defined above). This is indicated by the maximum percentage increase in cardiac parameters (***Q***, SV, SW) achieved at the maximum input pressure reached and by the number input pressure increments tolerated (Figure 1). Specifically, hearts removed from animals exposed to MgCl_2_ (1:1) or Mix 45′ showed the worst cardiac performance compared to hypothermia, Mix 20′, and MgCl_2_ (Figure 1).

Overall, on the basis of the Frank-Starling curves, the data suggest that magnesium chloride (1:1) and prolonged (45′) exposure to Mix should be avoided as agents for procedures where recovery of the animal is required, because of the sustained residual effect on the heart and should not be used if “normal” cardiac tissue is required for *in vitro* or molecular studies. In contrast, Mix 20′ and MgCl_2_ (3.5% sw, 20 min exposure) appear suitable formulations for anesthesia in cephalopods where recovery is required.

### What direct effects do the various formulations of MgCl_2_ have on cardiac function?

To investigate if the MgCl_2_ formulations used for anesthesia *in vivo* could have a direct effect on cardiac function, the systemic hearts were removed from animals killed under hypothermia and perfused with increasing concentrations of anesthetic solutions. The results show that, in contrast to ethanol alone, all the anesthetic formulations containing MgCl_2_ (sw, 1:1 and Mix) produced a concentration-related, acute onset bradycardia which at the higher concentrations arrested some hearts (Figure 3).

The bradycardia induced by the various MgCl_2_ formulation *in vitro* is consistent with reports that the heart rate is “very low” in *O. vulgaris* anesthetized with 3.5% MgCl_2_ in sea water (Fiorito et al., 2015) and “slow” in the same species anesthetized with MgCl_2_+EtOH (Grimaldi et al., 2013). Ethanol alone (from 0.125 to 1.0%) did not induce a significant bradycardia indicating that chronotropic effects of the MgCl_2_+EtOH mixture (i.e., MgCl_2_ 1.12 % + EtOH 1%) are not due to the ethanol. It should also be noted that the highest concentration of MgCl_2_ tested *in vitro* (2%) and which caused arrest in 50% of hearts was lower than the 3.5% commonly used for anesthesia *in vivo*. This may indicate that *in vivo* additional mechanisms such as ganglionic or central nervous system reflexes operate to protect the myocardium.

In addition to the bradycardia, a significant reduction in stroke volume was observed with the effects being more marked with MgCl_2_ (1:1) and Mix compared to the same concentration (1%) of MgCl_2_ in sea water (Figure 2). Again, comparison of the effects of Mix (1.12% + 1%) and ethanol alone (1%) shows that whilst there is a concentration related bradycardia with Mix (Figure 2), this is not the case with ethanol alone, although both Mix and ethanol can produce cardiac arrest (Figure 3).

It should be also noted that the highest concentration of ethanol we studied *in vitro* (1%) is lower than that commonly used (2–3%) to anesthetize cephalopods when used as the sole agent (review in Fiorito et al., 2015).

### Potential mechanisms involved in the cardiac effects of the MgCl_2_ anesthetic formulations

This study has established that three formulations of magnesium chloride used to anesthetize cephalopods affect the cardiac function and in the case of MgCl_2_ (1:1) and Mix 45′ we observed persistent effects. Investigation of mechanisms responsible was beyond the scope of this study but, for completeness, the most likely mechanisms are outlined below to provide pointers for future investigation.

#### Osmolality

We noted a difference in the osmolality of the magnesium chloride formulations (Table 1) used as anesthetic agents in cephalopods: MgCl_2_ (sw) and Mix being hypertonic, and MgCl_2_ (1:1) hypotonic, compared to sea water.

Haemolymph in Mediterranean *O. vulgaris* has an osmolality of ~1300 mOsm/Kg or higher (D'Aniello et al., 1986; Wells and Wells, 1989), therefore Mix (1.12/1%) and 2% MgCl_2_ in sw are relatively hypertonic, while MgCl_2_ (1:1) is hypotonic, and 1% ethanol in sw is isotonic. Our data do not allow us assessment of the magnitude of any contribution of osmolality alone to the cardiac effects observed, as hypo- (e.g., 2% MgCl_2_ 1:1), hyper- (e.g., 2% MgCl_2_ sw), and iso-tonic (e.g., Mix 0.56%/0.5%) MgCl_2_ formulations all produced a bradycardia and reduction in stroke volume.

Specific studies must be undertaken to test the effects of osmolality on the heart and in particular to ensure that the osmotic changes are not activating volume-regulated ion channels (see discussion in Deaton, 1997; Souza and Scemes, 2000) such as the TRPM3 member of the melastatin-like subfamily of the transient receptor potential (TRP) family activated by reduced extracellular osmolality (Grimm et al., 2003). Osmosensitive channels have not been reported in cephalopods as far as we are aware, but studies in the mammalian heart showing chloride currents activated by cell swelling and inhibited by cell shrinkage (Duan et al., 2000; Huang et al., 2009) suggest that the osmolality of the solutions may have some role in mediating the changes and should be investigated.

#### Magnesium

The well-known effects of elevated extracellular magnesium ion concentration in inducing bradycardia and cardiac arrest in mammals is consistent with the effects observed in this study. We estimate that the magnesium concentrations in the formulations are between ~2x (Mix 1.12%MgCl_2_/1%EtOH) and ~4x (3.5% MgCl_2_) those found in cephalopod haemolymph (D'Aniello et al., 1986; Brown and Lasek, 1990).

In mammals, cardiac effects of elevated magnesium ions are ascribed to depression of the sino-atrial node and atrio-ventricular conduction, caused by a direct effect of extracellular magnesium on transmembrane ionic current (Shine and Douglas, 1974; Specter et al., 1975) and/or an effect on sympathetic ganglia (Winkler et al., 1940; Engbaek, 1952; Dubé and Granry, 2003; Herroeder et al., 2011). In the octopus heart a putative pacemaker has been localized near the atrio-ventricular valves (Wells, 1983; Agnisola, 1994; Agnisola and Houlihan, 1994) and is a likely primary site at which MgCl_2_ produces bradycardia in cephalopods.

Magnesium ions are a physiological blocker of Ca^++^ channels (Iseri and French, 1984). In the mollusc *Mercenaria mercenaria* extracellular hypermagnesia negatively affects cardiac rhythmicity due to an effect Ca^++^ (Devlin, 1993) providing further support for a major role of magnesium ions in mediating the cardiac effects of the formulations used in this study.

#### Calcium, sodium, potassium, and chloride

Cephalopod haemolymph has a relatively high concentration of calcium ions (in *O. vulgaris* 19 mEq/L according to (D'Aniello et al., 1986)) similar to sea water (approximately 10.5 mM/L according to Robertson, 1953).

The 3.5% MgCl_2_ (1:1) formulation has approximately half the concentration of Ca^++^, Na^+^, K^+^, and Cl^−^ compared to sea water, whereas in the other two MgCl_2_ formulations utilized in this study (and presumably in all other published works) Ca^++^, Na^+^ and K^+^ concentrations are similar to sea water but Cl^−^ is higher.

Extracellular Ca^++^ plays a pivotal role in both autorhythmicity and cardiac muscle contractility in molluscs (Hill and Yantorno, 1979; Driedzic, 1985; Devlin, 1993; Gesser et al., 1997). In octopus both extracellular [Ca^++^] concentration (and thus trans-sarcolemmal Ca^++^-flux) and Ca^++^-release from the sarcoplasmic reticulum are crucial for systemic heart contraction (Gesser et al., 1997; Altimiras et al., 1999). Therefore, it is conceivable that the lower calcium concentration in the MgCl_2_ (1:1) formulation may compromise cardiac function. In addition, the effect of lowered Ca^++^ concentration is probably exacerbated by the presence of a relatively high concentration of Mg^++^ which further antagonizes the effects of Ca^++^ (see above).

We can only speculate about the effects on the heart of simultaneously reduced K^+^ and Cl^−^ in the 3.5% MgCl_2_ (1:1) formulation, but we would expect that reducing extracellular K^+^ would decrease excitability whereas reducing extracellular Cl^−^ would increase excitability.

The marked reduction in Na^+^ concentration is likely to reduce the amplitude of the action potential in any neural tissue in the heart or ganglia based upon studies of the effects of 50% reduction in Na^+^ concentration on the squid giant axon (Hodgkin and Katz, 1949).

Overall, whilst the effects of elevated magnesium ions on calcium fluxes in the heart provide the most likely explanation for the effects observed on stroke volume and heart rate, the contribution of osmolality and the altered concentrations of Ca^++^, Na^+^, K^+^, and Cl^−^ acting in concert requires direct investigation.

### Implications of the present study for anesthesia in cephalopods

Formulations of magnesium chloride utilized to anesthetise cephalopods cause bradycardia in the isolated heart. This is consistent with the descriptive reports of heart rate in anesthetized octopuses (e.g., Young, 1971a; see also: Andrews et al., 2013; Fiorito et al., 2015). A marked fall in heart rate together with the effects of magnesium chloride formulations on stroke volume will reduce cardiac output leading to a decrease in brain perfusion. The fall in haemolymph flow to the brain together with the marked reduction of ventilation is likely to lead to ischaemia of the brain (and other tissues) that could contribute to the anesthetic state induced by the various magnesium chloride formulations. Direct effects of the magnesium chloride formulations on the heart are likely to be compounded by the suppression of ventilation and muscular activity of the arms caused by anesthesia which contribute to venous return (Wells, 1983; King et al., 2005). In addition, acute hypoxia is itself associated with bradycardia and reduced aortic flow in cephalopods, as reported, for example, for *Nautilus pompilius* (Boutilier et al., 2000) and *O. vulgaris* (Wells, 1983). Furthermore, *in vivo* the anesthetic formulations may also have effects on the brain vasomotor lobe or peripherally on the cardiac ganglia.

Despite the effects of magnesium chloride formulations on ventilation and the heart, following anesthesia on returning an *O. vulgaris* to fresh sea water all externally assessed parameters return within ~30 min (review in Fiorito et al., 2015). Recovery from anesthesia is usually uneventful with octopus feeding within an hour (for review see Fiorito et al., 2015). However, marked suppression of cardiac function is an undesirable property of an anesthetic, particularly as in the case of the 3.5% MgCl_2_ (1:1 sw:dw) and prolonged exposure to MgCl_2_ and ethanol mixture (Mix 45′) where it is associated with residual deleterious cardiac effects.

In conclusion, this study has demonstrated for the first time the acute direct effects on cardiac function of three formulations of magnesium chloride used as anesthetics in cephalopods following Messenger et al. (1985). A direct effect of Mg^++^ on cardiac calcium fluxes can account for the marked bradycardia observed in animals anesthetized with MgCl_2_. Evidence was obtained for a residual effect of MgCl_2_ on cardiac function that could compromise recovery, but this was apparent only with either prolonged exposure (45 min) or using the sw:dw formulation.

Based upon the overall assessment of the acute (bradycardia/arrest and decreased stroke volume: Protocol II) and protracted (reduced Frank-Starling curves: Protocol I) effects, 3.5% MgCl_2_ in sea water and Mix formulations had the least deleterious combination of effects provided exposure time is minimized (within 30 min or less). These formulations may be suitable for procedures where relatively short duration (~20 min) anesthesia is required and for studies using non-invasive approaches (e.g., Grimaldi et al., 2007; Margheri et al., 2011).

In addition, these results pave the way for mechanistically oriented studies aimed to re-examine the most common anesthetic practices used with cephalopods in relation to the application of Directive 2010/63/EU.

## Ethics statement

No National Legislation was in place in Italy for regulation of research involving cephalopods when the studies were performed. Thus, they do not require approval by the national competent authority.

## Author contributions

CP, AG, RM, and MC designed the experiments; CP performed the experiments; CP, AG and RM collected and analyzed the data; GF and AG supervised CP during the relevant part of the PhD; MC providing also supervision and mentoring. GF and PA conceived the experiments. CP, AG, PA, and GF discussed the experimental design, data, and wrote the paper. All authors discussed the results and commented on the manuscript at all stages. All authors read and approved the submitted manuscript.

### Conflict of interest statement

The authors declare that the research was conducted in the absence of any commercial or financial relationships that could be construed as a potential conflict of interest.
